# Spatiotemporal responses of trabecular and cortical bone to complete spinal cord injury in skeletally mature rats

**DOI:** 10.1016/j.bonr.2022.101592

**Published:** 2022-05-21

**Authors:** Jonathan A. Williams, Carmen Huesa, James F.C. Windmill, Mariel Purcell, Stuart Reid, Sylvie Coupaud, John S. Riddell

**Affiliations:** a Department of Biomedical Engineering, Wolfson Building, University of Strathclyde, Glasgow G4 0NW, UK; b School of Neuroscience and Psychology, College of Medical, Veterinary and Life Sciences, University of Glasgow, G12 8QQ, UK; c Scottish Centre for Innovation in Spinal Cord Injury, Queen Elizabeth National Spinal Injuries Unit, Glasgow, UK; d Institute of Infection, Immunity and Inflammation, College of Medical, Veterinary and Life Sciences, University of Glasgow, G12 8QQ, UK; e Department of Electronic and Electrical Engineering, Royal College Building, University of Strathclyde, Glasgow G1 1XW, UK

**Keywords:** Spinal cord injury, Skeletally mature, Osteoporosis, microCT, Bone morphometry, Mechanical testing

## Abstract

**Objective:**

Characterise the spatiotemporal responses of trabecular and cortical bone to complete spinal cord injury (SCI) in the skeletally mature rat in the acute (4-week) period following injury.

**Methods:**

The spinal cord of 5-month old male rats was transected at the T9 level. Outcome measures were assessed using micro-computed tomography, three-point bending and serum markers at 1-, 2-, and 4-weeks post-transection. Comparison was made with time-0 and sham animals.

**Results:**

Lower levels of circulating serum bone formation markers and higher bone resorption markers suggested uncoupled bone turnover as early at 1-week post-transection. Micro-computed tomography showed metaphyseal and epiphyseal trabecular bone loss was observed only at 4-weeks post-transection. The bone loss was site-specific with a more severe reduction in trabecular BV/TV observed in the metaphyseal (50%) relative to epiphyseal (19%) region. Metaphyseal trabecular bone exhibited a 54% reduction in connectivity density while the epiphyseal trabecular bone was unaffected. Cortical bone deficits were not seen over the time periods examined.

**Conclusions:**

The study demonstrates that the skeletally mature spinal cord transected rat model replicates the biphasic pattern of osteoporotic changes observed in the human SCI population, providing a relevant model for testing the efficacy of interventions against SCI-induced osteoporosis.

## Highlights

• Skeletally mature spinal cord transected rats display biphasic bone loss
• The osteoporosis manifests over slower time scales than in skeletally immature rats.
• Relevancy for testing efficacy of interventions against SCI-induced osteoporosis.

## 1 Introduction

Studies in human spinal cord injury (SCI) populations have reported rapid loss of bone mass and severe deterioration of geometry in bones below the injury level, within the first few months and years of injury (Coupaud et al., 2015; Eser et al., 2004). The development of this induced osteoporosis occurs after complete SCI and significantly increases the risk of fragility fracture in the affected bones (Frotzler et al., 2015). Follow up studies suggest that approximately one half of the human SCI population will sustain at least one fragility fracture (Zehnder et al., 2004). The most common fracture sites are at the knee (proximal tibia and distal femur) (Frotzler et al., 2015). Over 50% of fractures come with further health complications, such as: modified healing resulting in delayed union, malunion or non-union of the fracture, the development of skin pressure ulcers, osteomyelitis, episodes of autonomic dysreflexia and heterotopic ossification (Sezer, 2015).

To improve osteoporosis management and reduce fracture risk in this patient population, appropriate interventions need to be tested. Whether the interventions are pharmacological, physical or biophysical, suitable pre-clinical animal models are needed to test their effectiveness. The animal model that has most frequently been used in this regard is the young (skeletally immature) rat model of SCI, whether the injury is produced via transection (Jiang et al., 2006, Jiang et al., 2007a, Jiang et al., 2007b; D. Liu et al., 2008a, Liu et al., 2008b; Minematsu et al., 2014, Minematsu et al., 2016; Peng et al., 2020; Williams et al., 2020, Williams et al., 2022) or contusion (Beggs et al., 2015; Morse et al., 2008). This is despite over 95% of spinal cord injuries occurring in adults (i.e. skeletally mature individuals) (Parent et al., 2011). It has been shown that the rapid deterioration of trabecular bone in the young, skeletally immature rat model of transection SCI resembles that observed in both adult and paediatric SCI, while cortical bone changes which are a combination of bone loss and supressed bone apposition better resemble that observed clinically in paediatric SCI only (Williams et al., 2022). For skeletally mature adult SCI rats, where bone modelling and bone growth have stopped, only one study has been performed that monitors bone changes at different time points post-SCI (Otzel et al., 2019). This was in a contusion model of SCI which results in a functionally incomplete injury where there can be substantial recovery of volitional hindlimb movement. The degree of recovery is dependent on injury severity, but usually includes weight supported stepping (Basso et al., 1996). This locomotor recovery confounds the study of paralysis-related bone loss (Lin et al., 2018). In contrast, paralysis resulting from transection of the spinal cord is both complete and permanent below the level of injury (Lilley et al., 2020), but to date, a time course study of bone loss has yet to be performed in skeletally mature rats using the transection model.

This study investigates the spatiotemporal responses of trabecular and cortical bone to complete T9-level transection SCI in the skeletally mature rat, over the acute period of injury (the first 4-weeks). The motivation is to assess the suitability of the model at replicating the clinical presentation of SCI-induced osteoporosis and in particular the time-frame over which SCI-induced changes occur.

The findings of this study have implications for the design of pre-clinical SCI-induced osteoporosis intervention studies, in particular for the start time of an intervention. Starting an intervention as soon as possible post-surgery enables the study of whether it can prevent/attenuate active bone loss, while delaying the intervention enables the study of whether it can reverse/ameliorate existing bone loss.

## 2 Material and methods

### 2.1 Animals

Forty-eight 10-week-old male Sprague-Dawley rats were acquired from Charles River Laboratories, Kent, UK. Rats were housed in threes, in a temperature-controlled room under a 12-h light-dark cycle, with *ad libitum* access to food and water, for 11-weeks. All experimental procedures were approved by the Ethical Review Panel of the University of Glasgow and carried out in accordance with the Animals (Scientific Procedures) Act 1986.

### 2.2 Experimental design

At 5-months (21-weeks) of age, rats were randomly assigned into SCI (*n* = 24), SHAM (*n* = 18) or Time-0 (*n* = 6) groups, where Time-0 rats were euthanised close to the time that surgery was performed on SCI and SHAM rats. Subsets of SCI and SHAM rats were euthanised at 1-, 2-, and 4-weeks post-surgery (*n* = 8 per SCI group, *n* = 6 for SHAM per group). Euthanasia was by anaesthetic overdose (Euthatal, Merial Animal Health Ltd., Harlow, UK). Blood samples were acquired at the time of sacrifice via the descending aorta. Serum was separated and stored at −80 °C. Right gastrocnemius muscles (both medial and lateral heads) and femora were dissected and weighed. Right femoral length was measured parallel to the shaft between the femoral head and condyles using digital callipers. Right femurs were wrapped in PBS-soaked gauze and stored at −20 °C for later micro-computed tomography (μCT).

### 2.3 Surgery and postoperative care

Surgery and postoperative care were carried out as previously described (Williams et al., 2020). Briefly, laminectomy was performed at the T9-T10 level in rats anesthetised with isoflourane. In SHAM rats, the spinal cord was exposed but the wound was then immediately stitched closed. In SCI rats, a transection injury was produced by making an opening in the dura and cutting the spinal cord transversely at two locations, approximately 1 mm apart. Tissue between the spinal cord transections was removed by aspiration and completeness of transection confirmed visually. Rats received buprenorphine (0.05 mg/kg s.c.) and carprofen (5 mg/kg s.c.) the morning of and morning after surgery. Saline (3–5 ml s.c.) and enrofloxacin (5 mg/kg s.c.) were given for 3- and 7-days post-surgery for SHAM and SCI groups, respectively. Bladders of SCI rats were expressed 3-times per day until spontaneous voiding returned, between 10- and 15-days post-surgery. Body mass was measured daily for the first 2- and 3-weeks for SHAM and SCI rats, respectively, then weekly. Two rats from the 2-weeks SCI group were euthanised due to complications preceding the designated end point. They were not included in the following analysis.

### 2.4 micro-computed tomography

Right femurs were μCT scanned using a Bruker SkyScan 1172 scanner (Kontich, Belgium) with a Hamamatsu 80 kVp/100 μA X-ray tube at 10 μm isotropic voxel size, as previously described (Williams et al., 2020). Briefly, all scans were performed using the following settings: 70 kVp X-ray tube voltage, 100 μA X-ray tube current, 470 ms integration time, with 2 k camera resolution and 0.4° rotation step for a total of 180° with a 0.5 mm thick aluminium filter. The entire femur was captured with 4 subscans. Projection images were reconstructed using SkyScan NRecon software (Version 1.6.9.18, Kontich, Belgium) into cross-sectional images, with the following reconstruction parameters: ring-artifact correction = 13, beam hardening correction = 40%, smoothing = 2.

**Fig. 1:**
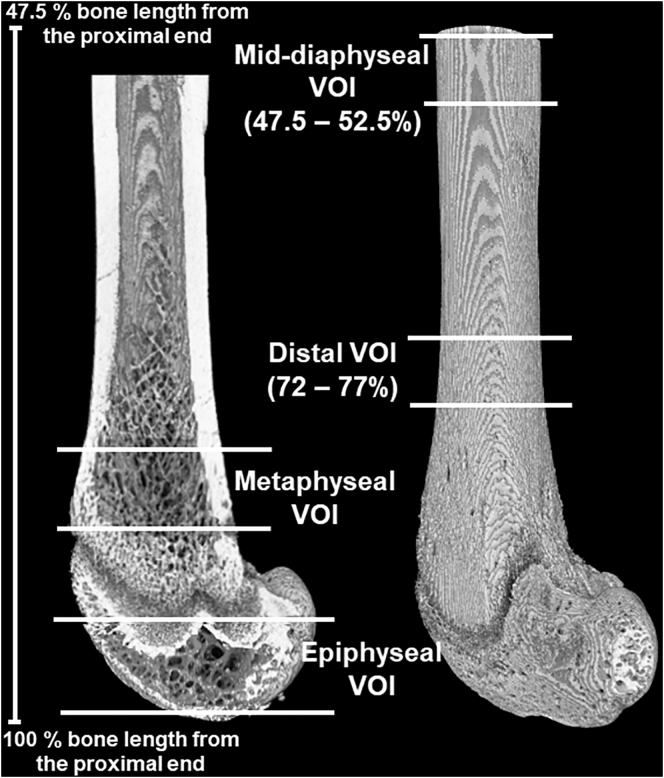
Schematic of the two trabecular and two cortical bone volumes of interest (VOIs) selected for 3D morphometric and densitometric analysis.

Four volumes of interest (VOIs) were selected for morphometric analysis: distal femoral epiphyseal trabecular bone, distal femoral metaphyseal trabecular bone, distal femoral cortical bone and mid-femoral diaphyseal cortical bone (Fig. 1). For the distal femoral epiphyseal region, all subchondral trabecular bone enclosed by the distal femoral growth plate was selected. A percentage-based selection approach was used for the remaining VOIs. The metaphyseal trabecular VOI started at a 2.5% bone length offset from the distal femoral growth plate reference slice, then extended into the metaphysis for a distance equal to 5% bone length. The distal and mid-diaphyseal cortical bone VOIs were located between 72 and 77%, and 47.5 and 52.5% bone length from the proximal end, respectively.

Epiphyseal trabecular bone was manually segmented from its thin enclosing cortical shell by a single operator. Metaphyseal trabecular and distal cortical bone VOIs were automatically segmented. Briefly, datasets were binarized using the Otsu segmentation algorithm (in 3D), followed by a despeckle operation (removal of all but the largest connected object, in 3D). The shrink-wrap operation (with stretch over holes with radius 32) was applied - this shrinks the region of interest (ROI) image to the boundaries (periosteum) of the bone. The exclusive or (XOR) was applied to the image and ROI producing a binarized image of the pore space only. Finally, sequential morphological opening and closing operations with a step wise increase in round kernel size from 2 to 16 voxels between sets was applied in 3D. This produces a consolidated binary image whose shape corresponds to that of the marrow cavity. Reloading the original grey scale dataset into this binary representation of the marrow cavity thus leads to the segmentation of trabecular bone from cortical bone. This technique is termed the morphological escalator. The mid-diaphyseal VOI was automatically segmented with single morphometric opening and closing operations. Subsequently, 3D morphometric analysis was performed on these VOIs after application of a Gaussian blur filter (σ = 1, kernel radius = 3, in 3D), binarisation (global threshold with lower limit set to 633 mgHA cm^−3^) and denoising (removal of white and black speckles (smaller than 75 voxels in 3D). Trabecular measures included: bone volume fraction (BV/TV), trabecular thickness (Tb.Th), trabecular number (Tb.N), trabecular separation (Tb.Sp), bone surface to bone volume ratio (BS/BV) and connectivity density (Conn.D) as per (Bouxsein et al., 2010) and trabecular pattern factor (Tb.Pf) (Hahn et al., 1992). Cortical measures included: cortical bone area (Ct.Ar), total area enclosed by the periosteum (Tt.Ar), marrow area (Ma.Ar), cortical thickness (Ct.Th), second polar moment of area (J) as per (Bouxsein et al., 2010) and eccentricity (Ecc) (Bruker Method Note). Trabecular volumetric bone mineral density (vBMD) and cortical tissue mineral density (TMD) were determined after calibration using two scanner manufacturer provided 4 mm diameter calibration hydroxyapatite phantoms, with known densities of 0.25 and 0.75 g cm^−3^. The CTAn task lists (.ctt files) used for automatic segmentation and morphometric analyses can be found in the online repository: https://github.com/JonathanAWilliams/Adult-Rat-Bone-SCI-Study-Scripts.

### 2.5 Three-point bend testing

Following μCT scanning, right femora underwent loading to failure in a three-point bend test. Femora were oriented in the anterior-posterior position (with the anterior surface in tension). The actuator head was lowered at a rate of 1 mm min^−1^, using a servohydraulic testing machine with a 2 kN load cell (Zwick/Roell z2.0, August-Nagel-Strasse 11, Ulm, Germany). The femora were preloaded to 10 N and allowed to adapt for 10 s before tested to failure. Load and actuator displacement were recorded at a sampling rate of 100 Hz, using testXpert II (Version 3.61) software. A 15 mm span length was used. The whole-bone structural properties; maximum load, stiffness and absorbed energy were obtained, and the tissue-level mechanical properties; elastic modulus and ultimate stress were calculated from the equations of beam theory (Turner and Burr, 1993). The whole-bone structural properties were extracted from the force-displacement graphs using a custom-made MATLAB script (Version 2019b). The MATLAB script can be found in the online repository: https://github.com/JonathanAWilliams/Adult-Rat-Bone-SCI-Study-Scripts.

### 2.6 Serum measurements

Bone formation and resorption serum markers were measured using Rat/Mouse Procollagen type 1 N-terminal propeptide (P1NP) and RatLaps™ C-terminal telopeptide of type I collagen (CTX) enzyme immunoassay kits (Immunodiagnostic Systems, Tyne & Wear, UK), respectively at time of death. Assays were performed following the manufacturer's instructions.

### 2.7 Statistics

Results are reported as means ± standard deviation (SD). Significance was defined as *p* < 0.05. A mixed-model repeated measures ANOVA was used to assess body mass at multiple time points within the same rats. Group (Time-0, SCI, SHAM) and time (1-, 2-, and 4-weeks post-surgery) main effects and interaction effects were determined with two-way [3 × 4] ANOVAs for all outcome measures, with Tukey's post hoc tests for multiple comparisons among groups. Additionally, three targeted Student's *t*-test for independent samples were performed *a priori* to determine differences between SCI and SHAM groups at the same post-surgery time point. All statistical analysis and plotting were performed using R (Version 3.6.1). These R scripts can be found in the online repository: https://github.com/JonathanAWilliams/Adult-Rat-Bone-SCI-Study-Scripts.

## 3 Results

**Fig. 2:**
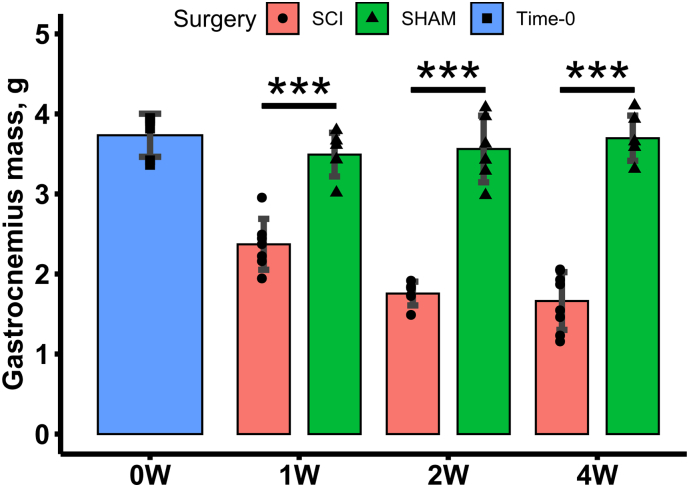
Gastrocnemius mass for Time-0, SCI and SHAM rats at 1-, 2- and 4-weeks post-surgery. Data shown as mean ± SD. *** indicate *p* < 0.001 for SCI versus SHAM at the same post-surgical timepoint.

Body mass at time of surgery was similar for Time-0, SCI and SHAM groups (Online Resource 1). From day 5 post-surgery and onwards, the body mass of the SCI group was lower than for SHAM animals (*p* < 0.05) (Online Resource 2). From day 4-post-surgery and onwards, the body mass of SCI animals declined compared to their weight at the time of surgery (p < 0.05), this is expected and is a result of the wasting of skeletal muscles below the level of injury (Online Resource 2). SHAM rats did not exhibit an increase in body mass relative to body mass at time of surgery over the 4-week period of this study (Online Resource 2). The gastrocnemius mass was significantly lower at all time points (1-, 2- and 4-weeks) post-surgery (*p* < 0.001) in SCI compared to SHAM groups (Fig. 2), whilst femoral mass and longitudinal length were unaffected by SCI (Online Resources 3 & 4). Neither SHAM or SCI groups exhibited growth-related increases in gastrocnemius or femoral mass or length, indicating that the animals were skeletally mature.

### 3.1 μCT analysis of trabecular bone morphometry and densitometry

**Fig. 3:**
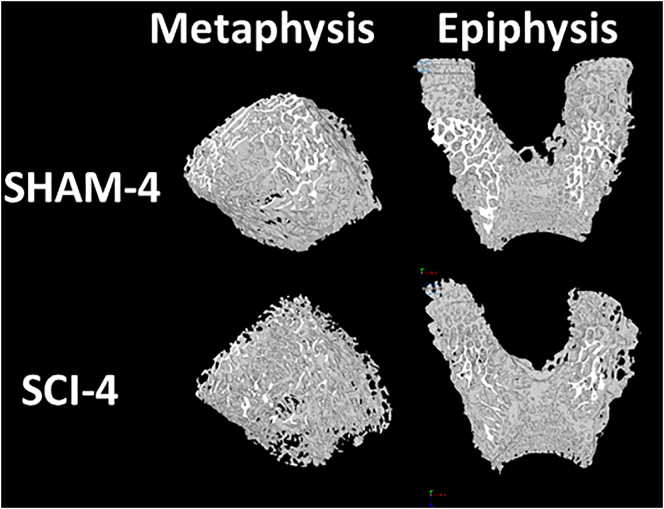
Representative μCT-based images of distal femoral metaphyseal and epiphyseal trabecular bone for 4-weeks post-surgery SHAM and SCI groups only. Representative images from the other groups not shown as they do not significantly differ in morphology from the 4-weeks post-surgery SHAM group.

**Fig. 4:**
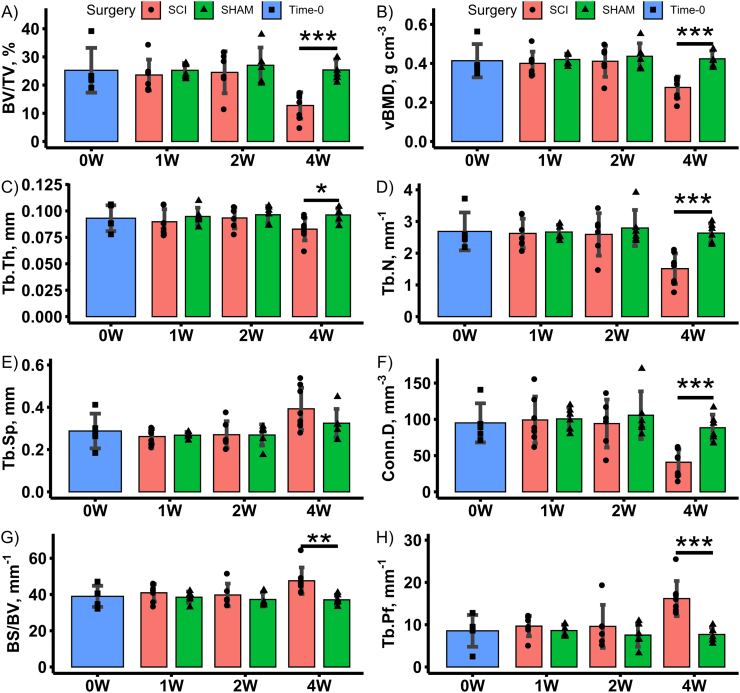
Mean morphometric and densitometric outcome measures for the distal femoral metaphyseal trabecular bone VOI for Time-0 and 1-, 2- and 4-weeks post-surgery SCI and SHAM groups. Data shown as mean ± SD. *, ** and *** indicate *p* < 0.05, *p* < 0.01 and *p* < 0.001, respectively, for SCI versus SHAM at the same post-surgical timepoint.

**Fig. 5:**
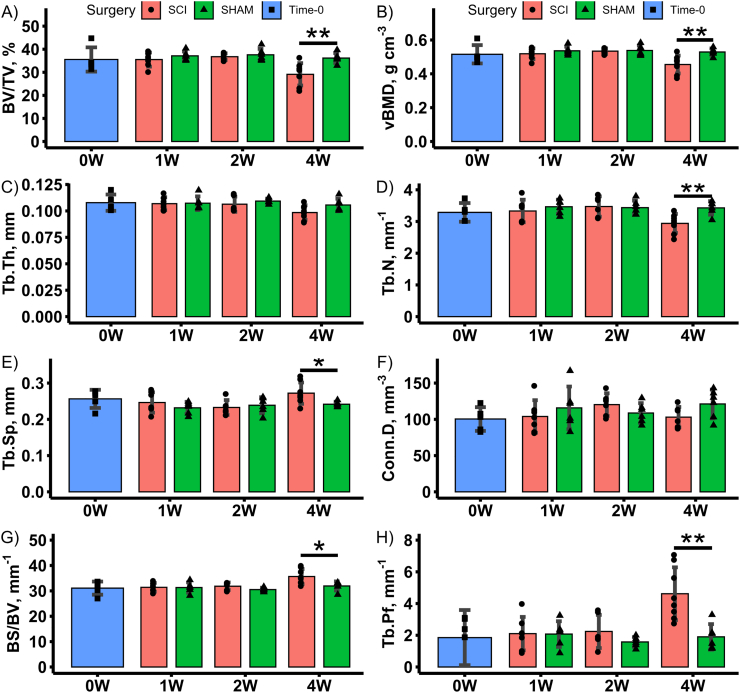
Mean morphometric and densitometric outcome measures for the distal femoral epiphyseal trabecular bone VOI for Time-0 and 1-, 2- and 4-weeks post-surgery SCI and SHAM groups. Data shown as mean ± SD. * and ** indicate p < 0.05 and p < 0.01, respectively, for SCI versus SHAM at the same post-surgical timepoint.

Representative VOIs for distal femoral metaphyseal and epiphyseal trabecular bone from a SHAM and SCI animal in the 4-week post-surgery groups are shown in Fig. 3. Mean morphometric and densitometric measures are shown for all groups in Fig. 4, Fig. 5. Group main effects indicated that SCI groups had lower distal femoral metaphyseal and epiphyseal trabecular BV/TV, vBMD, Tb.N (metaphysis only), Conn.D (metaphysis only) and higher Tb.Pf and BS/BV (all *p* < 0.05). Time main effects indicated lower BV/TV, vBMD, Tb.Th (epiphysis only), Tb.N, Conn.D (metaphysis only) and higher Tb.Sp, Tb.Pf and BS/BV (all *p* < 0.05). This suggests no progressive trabecular thickening, in contrast to observations in skeletally immature transected rats (Williams et al., 2022). Interaction effects revealed that the 4-weeks post-surgery SCI group had lower trabecular BV/TV (except Time-0 epiphysis), vBMD (except Time-0 epiphysis), Tb.Pf, Tb.N (metaphysis only) and Conn.D (metaphysis only), when compared to all other groups (all *p* < 0.05). Online Resource 5 contains a full analysis of main effects and interaction effects.

Targeted *t*-tests indicated that, at 4-weeks post-surgery, distal femoral metaphyseal and epiphyseal trabecular BV/TV were 50% (*p* < 0.001) and 19% lower (*p* < 0.01), respectively, in SCI compared with time-matched SHAM animals. The metaphyseal trabecular change was characterised by a 43% (p < 0.001) and 14% (*p* < 0.05) lowering of Tb.N and Tb.Th, respectively, and 28% (*p* < 0.01) higher BS/BV, in SCI animals. These structural changes led to 54% (*p* < 0.001) lower Conn.D and 110% higher (p < 0.001) Tb.Pf, in SCI animals. The epiphyseal trabecular change was characterised by 14% (*p* < 0.01) lower Tb.N and 13% and 12% (both p < 0.05) higher Tb.Sp and BS/BV, respectively, in SCI animals. These structural changes led to 143% (p < 0.01) higher Tb.Pf, in SCI animals. Metaphyseal and epiphyseal trabecular vBMD were 35% (p < 0.001) and 14% lower (p < 0.01) in the 4-week post-surgery SCI group compared to SHAM animals.

### 3.2 μCT analysis of cortical bone morphometry and densitometry

Group main effects indicated that SCI groups exhibited lower distal cortical area compared to SHAM groups (Online Resource 5). Time main effects indicated no significant differences in measured cortical bone morphometric or densitometric measures, suggesting no progressive cortical thickening, which is observed in skeletally immature transected rats (Williams et al., 2022), an no cortical thinning as observed in the chronic phase of bone loss after human SCI (Eser et al., 2004). Targeted *t*-tests indicated no differences in cortical morphology or densitometry for the mid-diaphyseal or distal cortical bone VOIs between SCI and SHAM animals at any post-surgical timepoint (Table 1, Table 2).

**Table 1:** Distal femoral metaphyseal (72–77% bone length) cortical bone morphology and densitometry for Time-0, SCI and SHAM rats at 1-, 2- and 4-weeks post-surgery.

Distal	Ct.Ar (mm^2^)	Tt.Ar (mm^2^)	Ma.Ar (mm^2^)	Ct.Th (mm)	J (mm^4^)	Ecc	TMD (g cm^−3^)
Time-0	7.24 ± 0.54	18.49 ± 0.47	11.25 ± 0.49	0.50 ± 0.03	36.55 ± 3.01	0.75 ± 0.02	1.176 ± 0.016
SCI-1	7.68 ± 0.77	20.00 ± 1.16	12.33 ± 0.78	0.52 ± 0.04	41.89 ± 6.14	0.73 ± 0.05	1.186 ± 0.010
SHAM-1	7.81 ± 0.85	20.08 ± 2.15	12.27 ± 1.90	0.52 ± 0.05	43.16 ± 7.79	0.75 ± 0.02	1.184 ± 0.009
SCI-2	7.31 ± 0.48	19.20 ± 2.16	11.89 ± 1.97	0.50 ± 0.04	38.10 ± 6.51	0.72 ± 0.04	1.195 ± 0.012
SHAM-2	7.84 ± 0.51	18.63 ± 1.95	10.79 ± 1.76	0.54 ± 0.05	40.26 ± 6.06	0.75 ± 0.05	1.192 ± 0.007
SCI-4	6.90 ± 1.10	18.35 ± 3.19	11.45 ± 2.39	0.49 ± 0.05	36.49 ± 11.62	0.71 ± 0.05	1.200 ± 0.024
SHAM-4	7.90 ± 0.43	20.06 ± 1.62	12.15 ± 1.51	0.53 ± 0.03	43.17 ± 5.44	0.75 ± 0.03	1.197 ± 0.014

Data expressed as mean ± SD. Ct.Ar, Cortical bone area; Tt.Ar, Total area enclosed by the periosteum; Ma.Ar, Marrow area; Ct.Th, Cortical bone thickness; J, Second polar moment of area; Ecc, Eccentricity; TMD, tissue mineral density.

**Table 2:** Mid-diaphyseal (47.5–52.5% bone length) cortical bone morphology and densitometry for Time-0, SCI and SHAM rats at 1-, 2- and 4-weeks post-surgery.

Mid-Diaphysis	Ct.Ar (mm^2^)	Tt.Ar (mm^2^)	Ma.Ar (mm^2^)	Ct.Th (mm)	J (mm^4^)	Ecc	TMD (g cm^−3^)
Time-0	8.43 ± 0.92	13.66 ± 1.78	5.22 ± 0.95	0.82 ± 0.06	26.60 ± 6.44	0.67 ± 0.04	1.265 ± 0.012
SCI-1	9.26 ± 0.91	15.13 ± 1.53	5.87 ± 0.86	0.84 ± 0.06	32.21 ± 6.26	0.63 ± 0.07	1.260 ± 0.004
SHAM-1	9.20 ± 0.92	14.75 ± 1.84	5.55 ± 1.02	0.86 ± 0.05	31.50 ± 7.09	0.66 ± 0.03	1.266 ± 0.009
SCI-2	8.70 ± 0.69	13.99 ± 1.67	5.29 ± 1.22	0.83 ± 0.06	27.63 ± 6.03	0.68 ± 0.04	1.268 ± 0.014
SHAM-2	9.07 ± 0.53	14.32 ± 1.43	5.25 ± 1.10	0.87 ± 0.07	29.70 ± 4.82	0.67 ± 0.03	1.267 ± 0.007
SCI-4	9.07 ± 1.19	14.42 ± 2.00	5.35 ± 0.95	0.85 ± 0.07	29.98 ± 7.79	0.64 ± 0.06	1.265 ± 0.030
SHAM-4	9.10 ± 0.59	14.66 ± 0.84	5.57 ± 0.30	0.87 ± 0.03	30.50 ± 3.54	0.65 ± 0.02	1.260 ± 0.025

Data expressed as mean ± SD. Ct.Ar, Cortical bone area; Tt.Ar, Total area enclosed by the periosteum; Ma.Ar, Marrow area; Ct.Th, Cortical bone thickness; J, Second polar moment of area; Ecc, Eccentricity; TMD, tissue mineral density.

### 3.3 Three-point bend testing

**Fig. 6:**
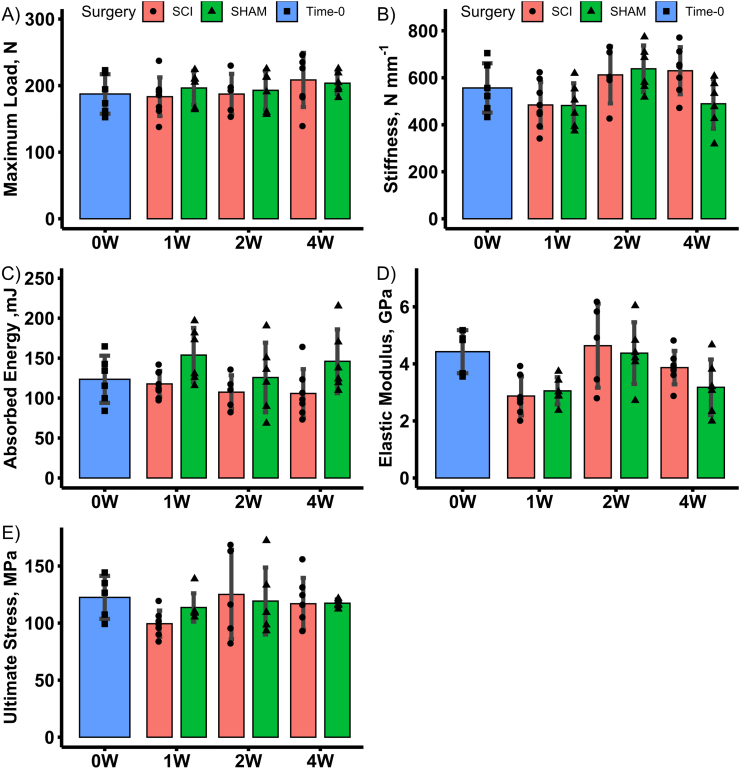
Three-point bend-determined whole-bone and material-level mechanical properties for Time-0 and 1-, 2- and 4-weeks post-surgery SCI and SHAM groups. Data shown as mean ± SD.

Group main effects indicated that SCI groups had lower absorbed energy to fracture compared to SHAM groups (*p* < 0.01) (Online Resource 5). Time main effects indicated increased stiffness and elastic modulus (both p < 0.01). There were no interaction effects. Targeted *t*-tests indicated no differences in whole-bone or material-level mechanical properties at any post-surgical timepoint (Fig. 6).

### 3.4 Blood serum analysis

**Fig. 7:**
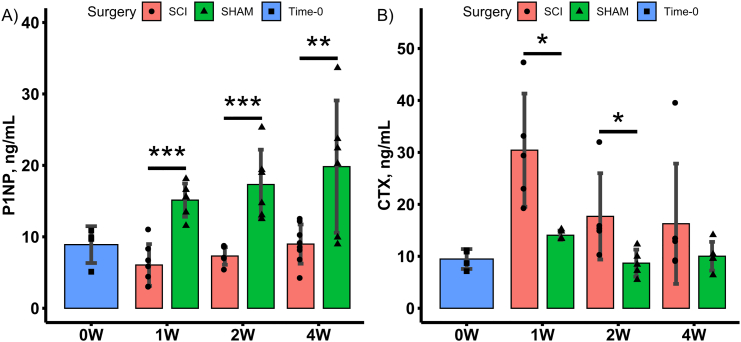
Serum bone turnover marker levels for Time-0, 1-, 2- and 4-weeks post-surgery SCI and SHAM groups. Data shown as mean ± SD. *, ** and *** indicate p < 0.05, *p* < 0.01 and p < 0.001, respectively for SCI versus SHAM at the same post-surgical timepoint. A) bone formation marker P1NP. B) bone resorption marker CTX.

Group main effects indicated that SCI had lower P1NP and higher CTX (both *p* < 0.001) (Online Resource 5). Time main effects indicate lower CTX for SCI (*p* < 0.05). Targeted *t*-tests revealed lower P1NP levels at all time points post-surgery while CTX levels were higher at 1- and 2-weeks post-surgery only, for SCI compared to SHAM (Fig. 7).

## 4 Discussion

In the clinical situation the vast majority of patients with SCI are skeletally mature adults (95%, Parent et al., 2011), and the majority of these male (80%, National Spinal Cord Injury Statistical Center, 2018). These individuals undergo an exponential decline in vBMD, particularly in the trabecular bone of the proximal tibial and distal femoral epiphyses (e.g. for the distal femur *VBMD* = 129.2*e*^−0.56*t*^ + 112.3, t = time in years) (Dudley-Javoroski and Shields, 2012; Eser et al., 2004). In contrast, cortical bone changes manifest over a longer period of time via decreased cortical vBMD associated with enlarged marrow cavities (endocortical thinning) (Dudley-Javoroski and Shields, 2012; Eser et al., 2004; Rittweger et al., 2010). These osteoporotic changes lead to an elevated fragility fracture risk, that increases with time after injury when compared to the population at large (Frotzler et al., 2015).

In this study, SCI was modelled in skeletally mature (5-month-old) male rats by complete transection of the spinal cord at the T9 level. Skeletal maturity was indicated by the observations that both longitudinal and appositional bone growth (measured by longitudinal length and total volume enclosed by the periosteum) had ceased (Online Resource 4, Table 1, Table 2). The time course effects of spinal cord transection on serum bone turnover markers indicated decreased circulating levels of the bone formation marker P1NP and increased levels of bone resorption marker CTX over the first 2-weeks post-surgery (Fig. 7). This is comparable to the uncoupled bone turnover reported acutely in the human SCI population (Reiter et al., 2007). Despite elevated resorption and suppressed formation markers as early as 1-week post-surgery, the concomitant distal femoral trabecular bone loss and accompanied microarchitectural deterioration was temporally delayed, with lower trabecular vBMD, BV/TV and deteriorated microarchitecture only detectable in 4-week post-surgery groups. A deficit in distal cortical bone area (Ct.Ar) and absorbed energy to fracture was observed as a group (SCI, SHAM) main effect (Online Resource 5). However targeted *t*-tests, comparing SHAM and SCI groups at individual time points studied (1-, 2- and 4-weeks post-surgery), showed no statistical difference in the morphometric or whole-bone mechanical properties. This minimal cortical bone loss at early time points in the rat model, is similar to the biphasic pattern of bone loss observed in human SCI populations, where trabecular bone loss precedes cortical bone loss (Frotzler et al., 2008).

In comparison to the delayed trabecular bone loss reported here (>2 weeks post-surgery), a recent study in skeletally immature (9-week-old) T3-T4 spinal cord transected rats observed that bone mass and microarchitecture were significantly deteriorated as early as 2-days post-surgery with concomitantly increased osteoclastic activity measured by circulating CTX and gene expression (Peng et al., 2020). This agrees with our previous work in skeletally immature low thoracic (T9) spinal cord transected rats, where a 59% lowering of metaphyseal trabecular BV/TV was observed at 2-weeks post-surgery compared to age-matched SHAM group (Williams et al., 2022). We also showed that epiphyseal trabecular bone (the trabecular bone most commonly scanned with peripheral quantitative computed tomography in the human SCI population) was subject to continued growth-related phenomena, which is not present in the skeletally mature model studied here. The cortical bone response in skeletally immature rats was manifested as more slender long bones (reduced Tt.Ar opposed to unchanged Tt.Ar and increased Ma.Ar), which is how SCI-induced osteoporotic bone changes present themselves in the paediatric population (Biggin et al., 2013). The comparison of SCI-induced osteoporosis in the skeletally mature rat reported here, with that of the human SCI population, highlights that the mature model closely reproduces the clinical situation. This is in contrast to our observations in the skeletally immature spinal cord transected rat (Williams et al., 2022). Transection injuries in skeletally mature rats should therefore be preferred when investigating SCI-induced-osteoporosis, unless paediatric SCI is the main interest.

There has only been one previous time course study of SCI-induced osteoporosis in skeletally mature rats (Otzel et al., 2019). This study used the moderate-severe contusion model where there is always recovery of motor function. In particular, 16-week-old male rats were subjected to a T9-contusion injury and groups of rats assessed at 2-weeks, 1-, 2- and 3-months post-surgery. Interestingly, significant trabecular bone loss was observed at the earliest time point assessed, while cortical bone deficits started to emerge in the 1- and 2-month post-surgery groups. Despite temporal disagreements in the emergence of SCI-induced osteoporosis, both skeletally mature contusion and transection models agreed well in the overall characteristic presentation at the 4-week/1-month time point post-surgery. Temporal differences in the presentation of osteoporotic bone changes may be accounted for by the age differences in rats used, with our rats being 5-weeks older.

There are other animal models which interfere with the normal use of the hindlimbs for locomotion that are reported to show predictable patterns of hindlimb bone loss. Hindlimb/tail suspension and limb taping/casting (Jee and Yao, 2001) prevent the hindlimbs from being used for weight support but do not prevent voluntary muscle contraction and do not deprive the tissues (including bone) of their normal nerve innervation. Other models completely prevent contraction of selected muscle groups. Sciatic nerve transection produces flaccid paralysis (no muscle contraction is possible) of the muscles supplied by branches of this nerve (mainly ankle muscles and knee flexors) (Jiang et al., 2006), while botulinum toxin A produces paralysis limited to those muscle into which the toxin is injected (Thomsen et al., 2012). Neither of these prevent weight supported stepping because other muscles used for locomotion (e.g. hip muscles and knee extensors) are not affected. Both of these models interrupt the nerve supply to part of the hindlimb and this may include bone. The spinal cord transection model used here results in complete and permanent paralysis of the whole of the hindlimbs (i.e. abolishes voluntary contraction in muscle at all joints) and therefore permanently prevents their use for weight support. The muscles are not denervated. They remain connected to spinal cord circuits and they may contract in response to reflex or spontaneous activity from the spinal cord. Reflex activity becomes enhanced in the animal model as in SCI patients (Adams and Hicks, 2005).

The various models involving hindlimb immobilisation therefore differ in the extent to which hindlimb muscles acting at different joints are affected, the degree to which hindlimb use in locomotion and posture is prevented, the duration of disuse and whether or not the nerve supply to the bone is directly interrupted. The spinal cord transection model is the only model that replicates the conditions (neuronal, hormonal, biomechanical) that exist in SCI. While similar patterns of bone loss are observed in each of the immobilisation models described above, the extent of bone loss differs and is less than we report here for spinal cord injury. There has been only one direct comparison of spinal cord transection with an immobilisation model. Jiang et al. (2006) showed that bone loss following sciatic nerve transection (neuroectomy) was less severe than following spinal cord transection, in the same study, with animals of the same age. This is most likely because sciatic nerve transected animals continue to use their hindlimbs for weight bearing and locomotion.

In addition to closely reflecting the SCI-induced osteoporosis observed in the human SCI population, the skeletally mature spinal cord transection model has several other advantages for the study of SCI-induced osteoporosis. Firstly, the hindlimb paralysis and concomitant cessation of sensation below the level of injury means that this model is suited for testing non-pharmacological interventions that require the attachment of a device directly to the hindlimb to deliver a targeted (as opposed to whole body) stimulus for long periods. The application of these interventions (e.g. vibration or electrical stimulation (Wu et al., 2013)) would usually require use of anaesthesia (Garman et al., 2007; Manske et al., 2012). In contrast, the contusion model is best suited for testing locomotor-type interventions (Yarrow et al., 2020) without robotic assistance for musculoskeletal recovery. Secondly, the time point post-SCI when an intervention is started is critical. The observation made here, that SCI-induced trabecular bone changes do not manifest until after 2-weeks post-surgery, make this a preferable model for testing non-pharmacological interventions in general, since before the intervention can start the rat must have sufficiently recovered from the severe surgery. At a minimum this takes 3-days, over which significant bone loss has already occurred in skeletally immature transected rats (Peng et al., 2020).

The use of ex vivo as opposed to in vivo μCT in this study is a limitation. In vivo μCT would allow for longitudinal analysis of SCI-induced osteoporosis in the same rats at multiple time points, not only increasing the pertinence of the results but also reducing the number of animals used in research. Additionally, dynamic histomorphometry for the measure of bone formation parameters and TRAP staining for resorption activity would have been useful additional modalities for further investigating the cellular response of SCI-induced osteoporosis.

To conclude, the osteoporosis induced by spinal cord transection in the skeletally mature male rat resembles the biphasic changes observed in the adult human SCI population where trabecular bone loss occurs over a shorter timescale than cortical bone changes. This indicates that a skeletally mature rat model is a highly relevant model for testing the efficacy of interventions designed to attenuate or reverse SCI-induced osteoporosis.

## Declarations of competing interest

None.

## CRediT authorship contribution statement

**Jonathan A. Williams:** Conceptualization, Methodology, Software, Validation, Data curation, Formal analysis, Investigation, Project administration, Writing – original draft, Writing – review & editing. **Carmen Huesa:** Investigation, Validation, Writing – review & editing. **James F.C. Windmill:** Methodology, Writing – review & editing. **Mariel Purcell:** Funding acquisition, Writing – review & editing. **Stuart Reid:** Funding acquisition, Supervision, Writing – review & editing. **Sylvie Coupaud:** Conceptualization, Funding acquisition, Supervision, Writing – review & editing. **John S. Riddell:** Conceptualization, Methodology, Validation, Investigation, Funding acquisition, Supervision, Writing – review & editing.

## Appendix A Supplementary data

Supplementary material Image 1
