# Supplementary material for: Spatiotemporal responses of trabecular and cortical bone to complete spinal cord injury in skeletally mature rats
Source: Bone Rep. 2022 May 21;16:101592. doi: 10.1016/j.bonr.2022.101592 (PMC9142855; doi:10.1016/j.bonr.2022.101592)
Supplement: Supplementary file 1 — Supplementary material [file mmc1.docx]

**Supplemental Material**

**
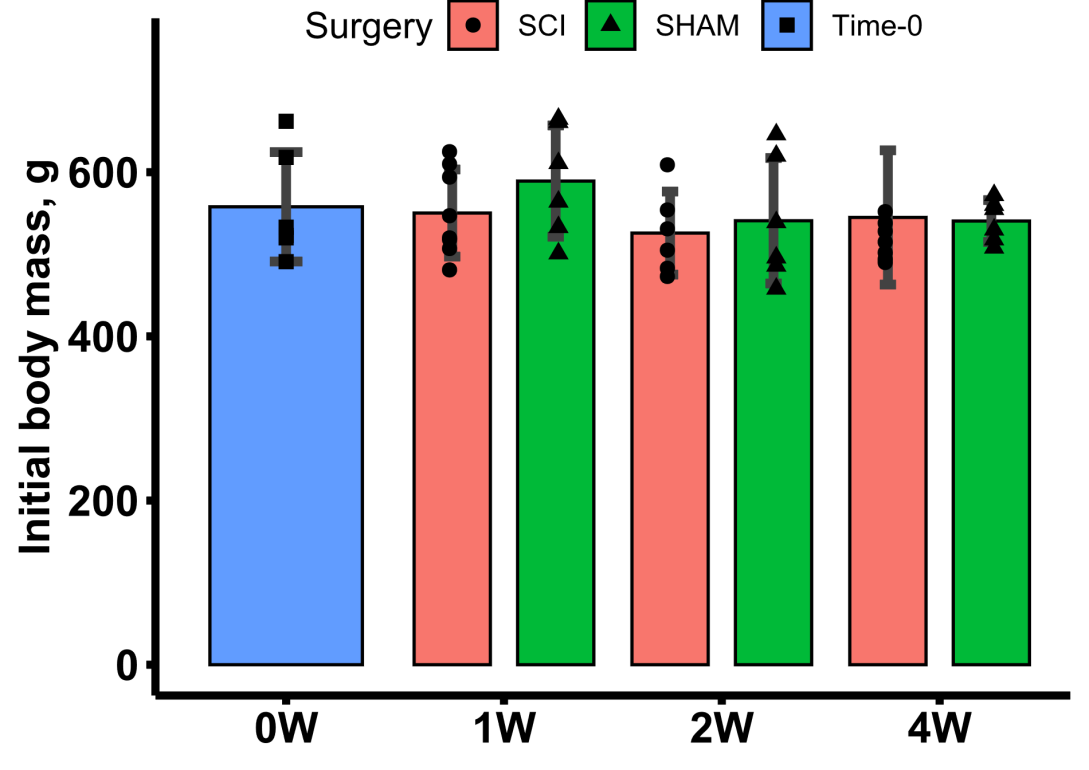
**

**Online Resource 1.** Body mass at time of surgery for all groups. Shown as mean ± SD. -1,-2 and -4 indicated 1-, 2- and 4-week post-surgery SHAM and SCI groups.


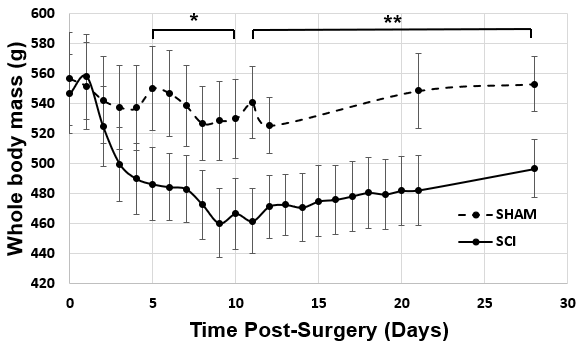


**Online Resource 2.** Body mass with time post-surgery for SCI and SHAM rats. Data shown as mean ± SD. * and ** indicate p < 0.05 and p < 0.01, respectively, for SCI versus SHAM at the same post-surgical timepoint.


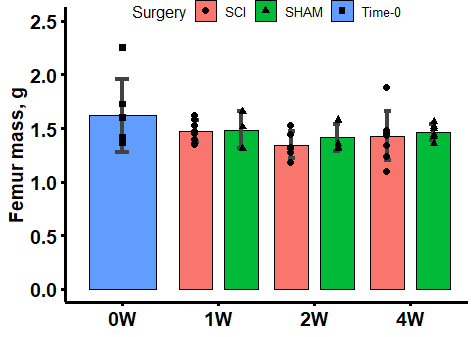


**Online Resource 3.** Femur wet mass for Time-0, SCI and SHAM rats at 1-, 2- and 4-weeks post-surgery. Data shown as mean ± SD.


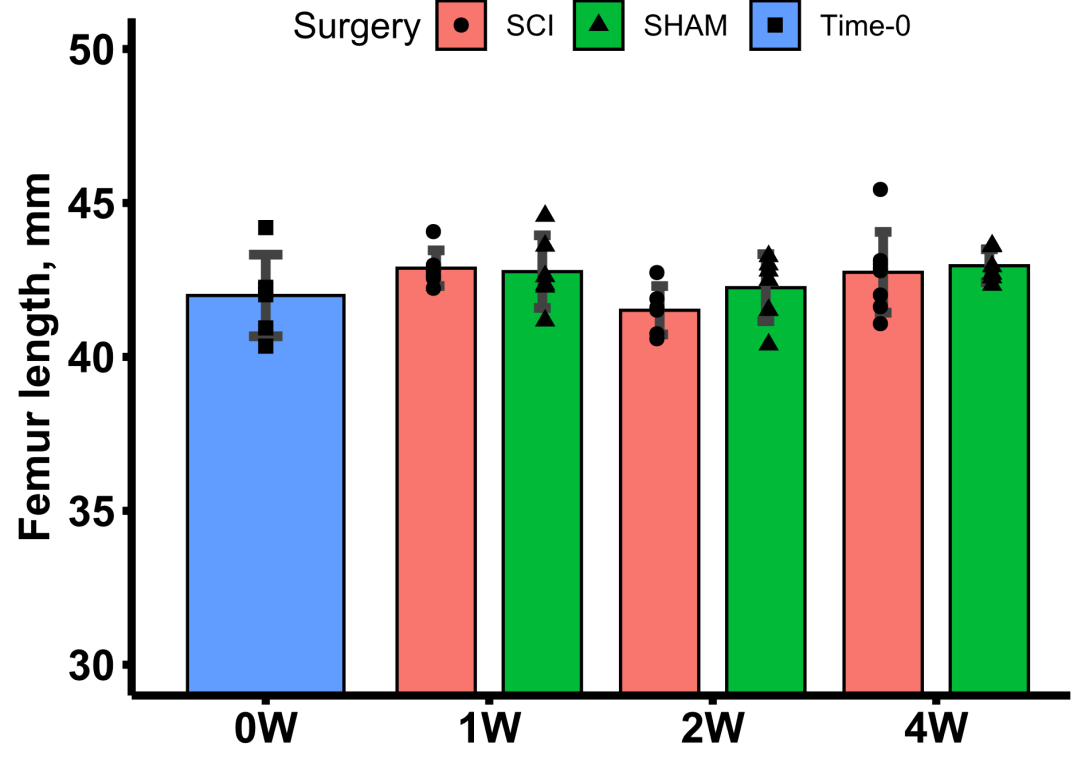


**Online Resource 4.** Femoral longitudinal length for Time-0, SCI and SHAM rats at 1-, 2- and 4-weeks post-surgery. Data shown as mean ± SD.

| **Online Resource 5.** Main effects and interactions from two-way ANOVA on bone and muscle wet weights, bone length, bone morphometric parameters and serum concentrations assessed at Time 0, 1-, 2- and 4-weeks post-surgery | | | | |
| --- | --- | --- | --- | --- |
| Bone and muscle wet weights and bone length | | | | |
| **Parameter** | **Main Effect** | **Main Effect** | **Interaction** | **Tukey Post Hoc** |
| **Gastrocnemius Mass (g)** | **Surgery: p = 2e-16*****  Time-0: 3.80 ± 0.11  SHAM: 3.58 ± 0.24  SCI: 2.23 ± 0.19 | **Time: p = 0.0265***  0 Week: 3.80 ± 0.11  1 Week: 3.38 ± 0.16  2 Week: 2.66 ± 0.18  4 Week: 2.68 ± 0.18 | **Interaction: p = 0.0015****  For Mean ± SD  See Fig 2 | Time 0 > SCI-1, SCI-2, SCI-4***  SHAM-1, SHAM-2, SHAM-4 > SCI-1***  SHAM-1 , SHAM-2, SHAM-4 > SCI-2***  SHAM-1, SHAM-2, SHAM-4 > SCI-4***  SCI-2 > SCI-1*  SCI-4 > SCI-1** |
| **Femur Mass (g)** | **Surgery: p = 0.0813**  Time-0: 1.62 ± 0.14  SHAM: 1.46 ± 0.09  SCI: 1.41 ± 0.10 | **Time: p = 0.3847**  0 Week: 1.62 ± 0.14  1 Week: 1.48 ± 0.08  2 Week: 1.38 ± 0.07  4 Week: 1.45 ± 0.09 | **Interaction: p = 0.9411**  For Mean ± SD  See Online Resource 3 | NS |
| **Femur Length (mm)** | **Surgery: p = 0.4075**  Time-0: 42.00 ± 0.60  SHAM: 42.67 ± 0.67  SCI: 42.37 ± 0.60 | **Time: p = 0.0681**  0 Week: 42.00 ± 0.60  1 Week: 42.85 ± 0.52  2 Week: 41.85 ± 0.55  4 Week: 42.85 ± 0.51 | **Interaction: p = 0.6003**  For Mean ± SD  See Online Resource 4 | NS |
| *µCT analysis of distal femoral metaphyseal trabecular morphology* | | | | |
| **Metaphyseal BV/TV (%)** | **Surgery: p = 0.0043****  Time-0: 25.3 ± 3.5  SHAM: 25.9 ± 3.2  SCI: 20.3 ± 4.0 | **Time: p = 0.0045****  0 Week: 25.3 ± 3.5  1 Week: 24.4 ± 2.3  2 Week: 25.8 ± 4.0  4 Week: 19.1 ± 2.2 | **Interaction: p = 0.287**  For Mean ± SD  See Fig 4A | Time-0, SCI-1, SCI-2 > SCI-4*  SHAM-1, SHAM-2, SHAM-4 > SCI-4** |
| **Metaphyseal vBMD (g cm^-3^)** | **Surgery: p = 0.0033****  Time-0: 0.41 ± 0.04  SHAM: 0.43 ± 0.03  SCI: 0.36 ± 0.04 | **Time: p = 0.0038****  0 Week: 0.41 ± 0.04  1 Week: 0.41 ± 0.03  2 Week: 0.42 ± 0.04  4 Week: 0.35 ± 0.03 | **Interaction: p = 0.0178***  For Mean ± SD  See Fig 4B | Time-0, SCI-1, SCI-2 > SCI-4*  SHAM-1, SHAM-2, SHAM-4 > SCI-4** |
| **Metaphyseal Tb.Th (mm)** | **Surgery: p = 0.0635**  Time-0: 0.093 ± 0.005  SHAM: 0.096 ± 0.005  SCI: 0.089 ± 0.007 | **Time: p = 0.3325**  0 Week: 0.093 ± 0.005  1 Week: 0.092 ± 0.006  2 Week: 0.095 ± 0.005  4 Week: 0.089 ± 0.005 | **Interaction: p = 0.3684**  For Mean ± SD  See Fig 4C | NS |
| **Metaphyseal Tb.N (mm^-1^)** | **Surgery: p = 0.0064****  Time-0: 2.69 ± 0.27  SHAM: 2.70 ± 0.28  SCI: 2.24 ± 0.37 | **Time: p = 0.0014****  0 Week: 2.69 ± 0.27  1 Week: 2.65 ± 0.20  2 Week: 2.69 ± 0.36  4 Week: 2.07 ± 0.21 | **Interaction: p = 0.0154***  For Mean ± SD  See Fig 4D | Time-0, SCI-1, SHAM-1, SHAM-2, SHAM-4 > SCI-4**  SCI-2 > SCI-4* |
| **Metaphyseal Tb.Sp (mm)** | **Surgery: p = 0.4139**  Time-0: 0.29 ± 0.04  SHAM: 0.29 ± 0.03  SCI: 0.31 ± 0.05 | **Time: p = 0.00051*****  0 Week: 0.29 ± 0.04  1 Week: 0.26 ± 0.01  2 Week: 0.27 ± 0.03  4 Week: 0.36 ± 0.04 | **Interaction: p = 0.2917**  For Mean ± SD  See Fig 4E | SHAM-1, SCI-1 < SCI-4* |
| **Metaphyseal Conn.D (mm^-3^)** | **Surgery: p = 0.0276***  Time-0: 95.1 ± 12.1  SHAM: 98.2 ± 16.6  SCI: 78.0 ± 19.3 | **Time: p = 0.00052*****  0 Week: 95.1 ± 12.1  1 Week: 99.9 ± 13.7  2 Week: 99.9 ± 19.1  4 Week: 64.6 ± 9.7 | **Interaction: p = 0.0680**  For Mean ± SD  See Fig 4F | Time-0, SCI-2 > SCI-4*  SHAM-1, SHAM-2, SCI-1 > SCI-4** |
| **Metaphyseal Tb.Pf (mm^-1^)** | **Surgery: p = 0.0010****  Time-0: 8.6 ± 1.7  SHAM: 8.0 ± 1.4  SCI: 11.8 ± 2.7 | **Time: p = 0.0126***  0 Week: 8.6 ± 1.7  1 Week: 9.1 ± 1.1  2 Week: 8.6 ± 2.4  4 Week: 11.9 ± 1.6 | **Interaction: p = 0.0121***  For Mean ± SD  See Fig 4H | Time-0, SCI-1, SCI-2 < SCI-4*  SHAM-1, SHAM-2, SHAM-4 < SCI-4** |
| **Metaphyseal BS/BV (mm^-1^)** | **Surgery: p = 0.0076****  Time-0: 39.0 ± 2.6  SHAM: 37.6 ± 2.4  SCI: 42.8 ± 4.1 | **Time: p = 0.1173**  0 Week: 39.0 ± 2.6  1 Week: 39.7 ± 2.2  2 Week: 38.5 ± 3.0  4 Week: 42.3 ± 2.8 | **Interaction: p = 0.0871**  For Mean ± SD  See Fig 4G | SHAM-2, SHAM-4 < SCI-4* |
| **Metaphyseal FD** | **Surgery: p = 0.0158***  Time-0: 2.56 ± 0.02  SHAM: 2.57 ± 0.03  SCI: 2.51 ± 0.05 | **Time: p = 0.00076*****  0 Week: 2.56 ± 0.02  1 Week: 2.58 ± 0.02  2 Week: 2.57 ± 0.03  4 Week: 2.48 ± 0.05 | **Interaction: p = 0.0325*** | Time-0, SCI-2, SHAM-4 > SCI-4*  SHAM-1, SHAM-2, SCI-1 > SCI-4** |
| *µCT analysis of distal femoral epiphyseal trabecular morphology* | | | | |
| **Epiphyseal BV/TV (%)** | **Surgery: p = 0.0088****  Time-0: 35.6 ± 2.4  SHAM: 37.0 ± 1.6  SCI: 33.8 ± 2.2 | **Time: p = 0.0015****  0 Week: 35.6 ± 2.4  1 Week: 36.3 ± 1.4  2 Week: 37.2 ± 1.2  4 Week: 32.6 ± 1.9 | **Interaction: p = 0.0449***  For Mean ± SD  See Fig 5A | SHAM-4, SCI-1 > SCI-4 *  SHAM-1, SHAM-2, SCI-2 > SCI-4 ** |
| **Epiphyseal vBMD (g cm^-3^)** | **Surgery: p = 0.0154***  Time-0: 0.52 ± 0.02  SHAM: 0.53 ± 0.02  SCI: 0.50 ± 0.02 | **Time: p = 0.0041****  0 Week: 0.52 ± 0.02  1 Week: 0.53 ± 0.02  2 Week: 0.54 ± 0.01  4 Week: 0.49 ± 0.02 | **Interaction: p = 0.0416**  For Mean ± SD  See Fig 5B | SHAM-1, SHAM-2 > SCI-4**  SHAM-4, SCI-2 > SCI-4* |
| **Epiphyseal Tb.Th (mm)** | **Surgery: p = 0.125**  Time-0: 0.108 ± 0.003  SHAM: 0.107 ± 0.004  SCI: 0.104 ± 0.004 | **Time: p = 0.033 ***  0 Week: 0.108 ± 0.003  1 Week: 0.107 ± 0.004  2 Week: 0.108 ± 0.003  4 Week: 0.102 ± 0.004 | **Interaction: p = 0.387**  For Mean ± SD  See Fig 5C | NS |
| **Epiphyseal Tb.N (mm^-1^)** | **Surgery: p = 0.0622**  Time-0: 3.29 ± 0.13  SHAM: 3.44 ± 0.14  SCI: 3.25 ± 0.22 | **Time: p = 0.0276 ***  0 Week: 3.29 ± 0.13  1 Week: 3.40 ± 0.16  2 Week: 3.45 ± 0.16  4 Week: 3.18 ± 0.14 | **Interaction: p = 0.0672**  For Mean ± SD  See Fig 5D | NS |
| **Epiphyseal Tb.Sp (mm)** | **Surgery: p = 0.0917**  Time-0: 0.26 ± 0.01  SHAM: 0.24 ± 0.02  SCI: 0.25 ± 0.01 | **Time: p = 0.0353***  0 Week: 0.26 ± 0.01  1 Week: 0.24 ± 0.01  2 Week: 0.24 ± 0.01  4 Week: 0.26 ± 0.01 | **Interaction: p = 0.1471**  For Mean ± SD  See Fig 5E | NS |
| **Epiphyseal Conn.D (mm^-3^)** | **Surgery: p = 0.277**  Time-0: 100.5 ± 7.3  SHAM: 115.2 ± 15.6  SCI: 109.1 ± 11.7 | **Time: p = 0.823**  0 Week: 100.5 ± 7.3  1 Week: 109.9 ± 14.7  2 Week: 114.5 ± 8.5  4 Week: 112.1 ± 9.5 | **Interaction: p = 0.145**  For Mean ± SD  See Fig 5F | NS |
| **Epiphyseal Tb.Pf (mm^-1^)** | **Surgery: p = 0.0045****  Time-0: 1.9 ± 0.8  SHAM: 1.9 ± 0.5  SCI: 3.0 ± 0.8 | **Time: p = 0.0043****  0 Week: 1.9 ± 0.8  1 Week: 2.1 ± 0.5  2 Week: 1.9 ± 0.5  4 Week: 3.3 ± 0.7 | **Interaction: p = 0.0136***  For Mean ± SD  See Fig 5H | Time-0, SHAM-2, SHAM-4, SCI-1 < SCI-4**  SCI-2, SHAM-1 < SCI-4* |
| **Epiphyseal BS/BV (mm^-1^)** | **Surgery: p = 0.0135***  Time-0: 31.1 ± 1.2  SHAM: 31.3 ± 1.2  SCI: 33.0 ± 1.4 | **Time: p = 0.0016****  0 Week: 31.1 ± 1.2  1 Week: 31.4 ± 1.1  2 Week: 31.2 ± 0.7  4 Week: 33.8 ± 1.3 | **Interaction: p = 0.811**  For Mean ± SD  See Fig 5E | Time-0, SCI-1, SHAM-1 < SCI-4*  SHAM-2 < SCI-4** |
| **Epiphyseal FD** | **Surgery: p = 0.0663**  Time-0: 2.54 ± 0.00  SHAM: 2.55 ± 0.01  SCI: 2.54 ± 0.01 | **Time: p = 0.0229***  0 Week: 2.54 ± 0.00  1 Week: 2.55 ± 0.01  2 Week: 2.55 ± 0.01  4 Week: 2.54 ± 0.01 | **Interaction: p = 0.0422*** | SHAM-1 > SCI-4* |
| *µCT analysis of distal femoral cortical morphology* | | | | |
| **Distal Tt.Ar**  **(mm^2^)** | **Surgery: p = 0.554**  Time-0: 18.5 ± 0.2  SHAM: 19.6 ± 1.4  SCI: 19.2 ± 1.5 | **Time: p = 0.342**  0 Week: 18.5 ± 0.2  1 Week: 20.0 ± 1.0  2 Week: 18.9 ± 1.2  4 Week: 19.2 ± 1.3 | **Interaction: p = 0.359**  For Mean ± SD  See Table 1 | NS |
| **Distal Ct.Ar**  **(mm^2^)** | **Surgery: p = 0.0481***  Time-0: 7.2 ± 0.2  SHAM: 7.9 ± 0.4  SCI: 7.3 ± 0.5 | **Time: p = 0.403**  0 Week: 7.2 ± 0.2  1 Week: 12.3 ± 0.8  2 Week: 11.3 ± 1.1  4 Week: 11.8 ± 1.1 | **Interaction: p = 0.322**  For Mean ± SD  See Table 1 | NS |
| **Distal Ma.Ar**  **(mm^2^)** | **Surgery: p = 0.771**  Time-0: 11.3 ± 0.22  SHAM: 11.7 ± 1.2  SCI: 11.9 ± 1.2 | **Time: p = 0.387**  0 Week: 11.3 ± 0.22  1 Week: 38.8 ± 2.0  2 Week: 40.4 ± 2.4  4 Week: 38.7 ± 1.8 | **Interaction: p = 0.421**  For Mean ± SD  See Table 1 | NS |
| **Distal Ct.Ar/Tt.Ar (%)** | **Surgery: p = 0.190**  Time-0: 39.2 ± 1.1  SHAM: 40.3 ± 2.7  SCI: 38.2 ± 2.3 | **Time: p = 0.450**  0 Week: 39.2 ± 1.1  1 Week: 38.8 ± 2.0  2 Week: 40.4 ± 2.4  4 Week: 38.7 ± 1.8 | **Interaction: p = 0.503** | NS |
| **Distal Ct.Th (mm)** | **Surgery: p = 0.108**  Time-0: 0.50 ± 0.02  SHAM: 0.53 ± 0.03  SCI: 0.50 ± 0.03 | **Time: p = 0.714**  0 Week: 0.50 ± 0.02  1 Week: 0.52 ± 0.03  2 Week: 0.52 ± 0.02  4 Week: 0.51 ± 0.02 | **Interaction: p = 0.464**  For Mean ± SD  See Table 1 | NS |
| **Distal BS/BV (mm^-1^)** | **Surgery: p = 0.0562**  Time-0: 5.9 ± 0.2  SHAM: 5.7 ± 0.3  SCI: 6.0 ± 0.3 | **Time: p = 0.658**  0 Week: 5.9 ± 0.2  1 Week: 5.8 ± 0.3  2 Week: 5.8 ± 0.3  4 Week: 6.0 ± 0.2 | **Interaction: p = 0.314** | NS |
| **Distal Ecc** | **Surgery: p = 0.107**  Time-0: 0.75 ± 0.01  SHAM: 0.75 ± 0.02  SCI: 0.72 ± 0.03 | **Time: p = 0.839**  0 Week: 0.75 ± 0.01  1 Week: 0.74 ± 0.02  2 Week: 0.74 ± 0.03  4 Week: 0.73 ± 0.02 | **Interaction: p = 0.927**  For Mean ± SD  See Table 1 | NS |
| **Distal J (mm^4^)** | **Surgery: p = 0.213**  Time-0: 36.6 ± 1.4  SHAM: 41.8 ± 4.6  SCI: 39.3 ± 5.4 | **Time: p = 0.452**  0 Week: 36.6 ± 1.4  1 Week: 42.5 ± 3.9  2 Week: 39.2 ± 3.6  4 Week: 39.8 ± 4.7 | **Interaction: p = 0.607**  For Mean ± SD  See Table 1 | NS |
| **Distal TMD (g cm^-3^)** | **Surgery: p = 0.0686**  Time-0: 1.176 ± 0.007  SHAM: 1.191 ± 0.007  SCI: 1.194 ± 0.011 | **Time: p = 0.0636**  0 Week: 1.176 ± 0.007  1 Week: 1.185 ± 0.005  2 Week: 1.193 ± 0.006  4 Week: 1.199 ± 0.010 | **Interaction: p = 0.995**  For Mean ± SD  See Table 1 | NS |
| *µCT analysis of diaphyseal cortical morphology* | | | | |
| **Diaphysis Tt.Ar**  **(mm^2^)** | **Surgery: p = 0.518**  Time-0: 13.7 ± 0.8  SHAM: 14.6 ± 1.0  SCI: 14.5 ± 1.1 | **Time: p = 0.480**  0 Week: 13.7 ± 0.8  1 Week: 14.9 ± 0.9  2 Week: 14.2 ± 0.9  4 Week: 14.5 ± 0.8 | **Interaction: p = 0.837**  For Mean ± SD  See Table 2 | NS |
| **Diaphysis Ct.Ar**  **(mm^2^)** | **Surgery: p = 0.299**  Time-0: 8.4 ± 0.4  SHAM: 9.1 ± 0.5  SCI: 9.0 ± 0.6 | **Time: p = 0.608**  0 Week: 8.4 ± 0.4  1 Week: 9.2 ± 0.5  2 Week: 8.9 ± 0.3  4 Week: 9.1 ± 0.5 | **Interaction: p = 0.804**  For Mean ± SD  See Table 2 | NS |
| **Diaphysis Ma.Ar**  **(mm^2^)** | **Surgery: p = 0.837**  Time-0: 5.2 ± 0.4  SHAM: 5.5 ± 0.6  SCI: 5.5 ± 0.7 | **Time: p = 0.492**  0 Week: 5.2 ± 0.4  1 Week: 5.8 ± 0.5  2 Week: 5.3 ± 0.7  4 Week: 5.5 ± 0.3 | **Interaction: p = 0.770**  For Mean ± SD  See Table 2 | NS |
| **Diaphysis Ct.Ar/Tt.Ar (%)** | **Surgery: p = 0.904**  Time-0: 62.3 ± 1.3  SHAM: 62.8 ± 2.3  SCI: 62.3 ± 2.4 | **Time: p = 0.659**  0 Week: 62.3 ± 1.3  1 Week: 61.9 ± 1.7  2 Week: 63.1 ± 2,7  4 Week: 62.5 ± 1.1 | **Interaction: p = 0.611** | NS |
| **Diaphysis Ct.Th (mm)** | **Surgery: p = 0.395**  Time-0: 0.82 ± 0.02  SHAM: 0.86 ± 0.04  SCI: 0.84 ± 0.04 | **Time: p = 0.999**  0 Week: 0.82 ± 0.02  1 Week: 0.85 ± 0.03  2 Week: 0.85 ± 0.04  4 Week: 0.85 ± 0.03 | **Interaction: p = 0.499**  For Mean ± SD  See Table 2 | NS |
| **Diaphysis BS/BV (mm^-1^)** | **Surgery: p = 0.228**  Time-0: 3.8 ± 0.1  SHAM: 3.6 ± 0.1  SCI: 3.7 ± 0.1 | **Time: p = 0.735**  0 Week: 3.8 ± 0.1  1 Week: 3.7 ± 0.1  2 Week: 3.7 ± 0.1  4 Week: 3.7 ± 0.1 | **Interaction: p = 0.557** | NS |
| **Diaphysis Ecc** | **Surgery: p = 0.647**  Time-0: 0.67 ± 0.02  SHAM: 0.66 ± 0.02  SCI: 0.65 ± 0.04 | **Time: p = 0.296**  0 Week: 0.67 ± 0.02  1 Week: 0.65 ± 0.03  2 Week: 0.67 ± 0.02  4 Week: 0.65 ± 0.02 | **Interaction: p = 0.603**  For Mean ± SD  See Table 2 | NS |
| **Diaphysis J (mm^4^)** | **Surgery: p = 0.453**  Time-0: 26.6 ± 2.9  SHAM: 30.6 ± 3.8  SCI: 29.9 ± 4.4 | **Time: p = 0.438**  0 Week: 26.6 ± 2.9  1 Week: 31.9 ± 3.8  2 Week: 28.7 ± 3.2  4 Week: 30.2 ± 3.1 | **Interaction: p = 0.857**  For Mean ± SD  See Table 2 | NS |
| **Diaphysis vBMD (g cm^-3^)** | **Surgery: p = 0.996**  Time-0: 1.265 ± 0.005  SHAM: 1.264 ± 0.011  SCI: 1.265 ± 0.012 | **Time: p = 0.730**  0 Week: 1.265 ± 0.005  1 Week: 1.263 ± 0.005  2 Week: 1.268 ± 0.006  4 Week: 1.263 ± 0.015 | **Interaction: p = 0.741**  For Mean ± SD  See Table 2 | NS |
| Three-point bend-determined whole-bone and material-level mechanical properties | | | | |
| **Maximum Load (N)** | **Surgery: p = 0.748**  Time-0: 187.4 ± 12.1  SHAM: 197.7 ± 17.1  SCI: 193.0 ± 22.9 | **Time: p = 0.268**  0 Week: 187.4 ± 12.1  1 Week: 189.9 ± 14.6  2 Week: 190.1 ± 18.1  4 Week: 206.1 ± 16.7 | **Interaction: p = 0.737**  For Mean ± SD  See Fig 6A | NS |
| **Stiffness N (mm^-1^)** | **Surgery: p = 0.656**  Time-0: 556.7 ± 42.9  SHAM: 536.5 ± 69.0  SCI: 575.4 ± 74.4 | **Time: p = 0.00389****  0 Week: 556.7 ± 42.9  1 Week: 483.1 ± 51.7  2 Week: 625.2 ± 67.6  4 Week: 559.6 ± 55.2 | **Interaction: p = 0.1104**  For Mean ± SD  See Fig 6B | NS |
| **Absorbed Energy (mJ)** | **Surgery: p = 0.0171***  Time-0: 123.5 ± 12.1  SHAM: 141.9 ± 27.2  SCI: 110.3 ± 16.0 | **Time: p = 0.3183**  0 Week: 123.5 ± 12.1  1 Week: 135.8 ± 15.0  2 Week: 116.6 ± 20.2  4 Week: 125.9 ± 19.0 | **Interaction: p = 0.6767**  For Mean ± SD  See Fig 6C | NS |
| **Elastic Modulus (GPa)** | **Surgery: p = 0.1537**  Time-0: 4.4 ± 0.3  SHAM: 3.5 ± 0.6  SCI: 3.8 ± 0.7 | **Time: p = 0.0006*****  0 Week: 4.4 ± 0.3  1 Week: 3.0 ± 0.3  2 Week: 4.5 ± 0.8  4 Week: 3.5 ± 0.4 | **Interaction: p = 0.4684**  For Mean ± SD  See Fig 6D | SCI-2 > SCI-1* |
| **Ultimate Stress (MPAO** | **Surgery: p = 0.638**  Time-0: 122.5 ± 8.4  SHAM: 116.8 ± 15.6  SCI: 113.8 ± 18.1 | **Time: p = 0.201**  0 Week: 122.5 ± 8.4  1 Week: 106.5 ± 6.7  2 Week: 122.2 ± 21.2  4 Week: 117.2 ± 8.6 | **Interaction: p = 0.517**  For Mean ± SD  See Fig 6E | NS |
| Blood serum bone formation and resorption markers | | | | |
| **P1NP (ng/mL)** | **Surgery: p = 1.47e-7*****  Time-0: 8.91 ± 1.29  SHAM: 17.40 ± 0.95  SCI: 7.45 ± 1.56 | **Time: p = 0.108**  0 Week: 8.91 ± 1.29  1 Week: 10.58 ± 1.46  2 Week: 12.31 ± 2.05  4 Week: 14.39 ± 3.90 | **Interaction: p = 0.877**  For Mean ± SD  See Fig 7A | SHAM-1 > SCI-1*  SHAM-2 > SCI-1**, SCI-2*  SHAM-4 > Time 0*, SCI-1***, SCI-2**,SCI-4** |
| **CTX (ng/mL)** | **Surgery: p = 0.0009*****  Time-0: 9.48 ± 0.96  SHAM: 10.93 ± 1.59  SCI: 21.50 ± 6.72 | **Time: p = 0.0107***  0 Week: 9.48 ± 0.96  1 Week: 22.25 ± 4.14  2 Week: 13.19 ± 3.55  4 Week: 13.20 ± 4.24 | **Interaction: p = 0.2822**  For Mean ± SD  See Fig 7B | SCI-1 > Time 0, SHAM-2, SHAM-4** |
